# VisualEars: How an immersive art exhibit impacts mood during the COVID-19 pandemic

**DOI:** 10.3389/fpsyg.2022.910767

**Published:** 2022-10-06

**Authors:** Shafagh Hadavi, Kody G. Kennedy, Geneva Mariotti, Joseph F. X. DeSouza

**Affiliations:** ^1^Graduate Program in Interdisciplinary Studies, York University, Toronto, ON, Canada; ^2^Department of Pharmacology, University of Toronto, Toronto, ON, Canada; ^3^Department of Psychology, York University, Toronto, ON, Canada; ^4^Centre for Vision Research, York University, Toronto, ON, Canada; ^5^Department of Biology, York University, Toronto, ON, Canada; ^6^Neuroscience Graduate Diploma Program, York University, Toronto, ON, Canada; ^7^Multisensory Neuroscience Laboratory, York University, Toronto, ON, Canada; ^8^VISTA & Canadian Action and Perception Network (CAPnet), University of Toronto, Toronto, ON, Canada

**Keywords:** visual art, music, mood enhancement, PANAS, virtual art exhibit, mental health, COVID-19 pandemic

## Abstract

This paper explores the positive impact of viewing a virtual art exhibit on mood during the COVID-19 Pandemic. During global lockdowns, depression, anxiety, and the burden of other mental illnesses have increased even among prior psychiatrically healthy individuals. Art and music-based interventions have shown to be effective clinical interventions in individuals with mental illness. The VisualEars project explored whether a virtual activity involving vision and auditory stimuli could improve positive and negative affect. Eight musical pieces were selected, and 28 visual artists from around the world visualized two musical pieces. A total of 56 works of art were created and hung in eight 3D virtual rooms. Visitors were randomly selected to either view the art exhibit without music (non-immersive) or view the art exhibit while listening to music (immersive). Visitors were asked to complete a positive and negative affect schedule (PANAS) in three languages (English, French, and Farsi) pre and post their virtual visit. A total of 160 participants completed baseline PANAS, 58 of which completed the follow-up PANAS. Linear mixed-effects models found that older participants had lower negative affect scores overall (*b* = −0.3, *p* = 0.003), while male participants had lower positive affect scores overall (*b* = −0.27, *p* = 0.02). Following the virtual exhibit participants of both conditions had higher positive (*b* = 0.17, *p* = 0.03), and lower negative affect scores (*b* = −0.19, *p* = 0.007). We found that the virtual art exhibit increased positive affect and decreased negative affect in participants, suggesting an overall improvement in mood attributable to the virtual exhibit. This suggests that virtual exhibits may serve as a beneficial and accessible intervention to improve mood during a pandemic.

## Introduction

The COVID-19 pandemic declared on March 11th, 2020, had a devastating impact on people’s physical and mental health across the globe. Sars-COVID 2 generated an infectious disease that took the lives of millions and infected around 1.5 to 2 billion people globally (Ioannidis, 2021). Governments introduced major lockdowns and tight restrictions to curb the spread and protect people from infecting one another. The anxiety of an unknown possibly deadly disease, fear of subsequent mutations, and the loss of jobs, family members, and inability to maintain a balanced life in addition to social isolations caused by the restrictions led to an increase in mental health problems such as major depressive disorder and anxiety disorders in many countries (COVID-19 Mental Disorders Collaborators, 2021COVID-19 Mental Disorders Collaborators, 2021). Technological solutions created opportunities for people to socialize, learn, and work virtually as much as possible. However, the ongoing speaking to a screen also resulted in other phenomena such as zoom fatigue (Bailenson, 2021). In the United States, surveys from spring 2020 to January 2021 showed 41% of adults reported symptoms of anxiety and/or depressive disorder. Additionally, 13% of adults reported new or increased substance use as a result of COVID-19-related stress, and in January 2021 11% of adults reported contemplating suicide in the past 30 days (Panchal et al., 2021). According to 6 surveys conducted by Leger for the Canadian Centre on Substance Use and Addiction and the Mental Health Commission of Canada on the Canadian youth and adult population from October 2020 to July 2021, mental health issues became more prevalent as a result of the pandemic. Youth (aged 16 to 24) reported more mental health and substance use concerns during the pandemic, and decreased ability to handle pandemic-related stress. Further, 45% of Youth reported moderate-severe anxiety. Substance use habits increased during the pandemic, with 40% of youth and 20% of older adults reporting an increase in use (Canadian Centre on Substance Use and Addiction & Mental Health Commission of Canada, 2021). This is a further concern as individuals with substance use concerns were reported to show signs of worsening mental health during the pandemic.

According to a study by the Canadian Centre on Substance Use and Addiction, only 22% of the surveyed population who showed symptoms of mental health issues received necessary treatment from March to December 2020 (Canadian Centre on Substance Use and Addiction & Mental Health Commission of Canada, 2021). Nonetheless, due to the continued stigma around mental health issues, it can be safely assumed that these numbers are much higher than reported in reality. Given the dire situation of mental health globally, adverse side effects from medications, and the stigmatization of mental disorders, this study aims to explore how immersive experiences such as those proposed by this study can serve as a potential alternative and highly affordable and accessible intervention for mood disturbances in the general population.

The visual arts’ positive benefits on mood and wellbeing have been used for centuries as powerful healing agents in many societies. Having visual works of art in early hospitals, churches and Buddhist shrines is believed to improve our wellbeing, and more specifically “bring about a change in consciousness and to promote healing and hope” (Samuels and Lane, 2013). This holistic approach supports the fact that mental and physical health are interconnected. Moreover, the significant role of emotions and thoughts have been widely discussed in health-related research (Sapolsky, 2000). A phenomenon called “The Museum Effect” has been reported in literature, and accounts for an individuals’ positive change in attitude toward other people while visiting an art exhibition (Smith, 2014).

The implications of visual arts in our surroundings have been documented in both clinical and non-clinical settings (Staricoff, 2004). Within the clinical setting, the environment in which the patients are placed in the hospital, and the amount of natural light and the scenery they are exposed to impacts their blood pressure, levels of stress and clinical outcomes as well as promote a safe place, connect them to the outside world, create an opportunity for socialization, and pain reduction (Frandsen et al., 2009; O’Bróin, 2015; Nielsen et al., 2017). Museums and the arts improve the wellbeing of communities through cultural contributions that can complement conventional medicine (White, 2009). In Germany, the ARTEMIS project (an art museum-based intervention) measured quality of life and mood in individuals with dementia, finding that they had improved quality of life with a decrease in negative affects such as depression and anxiety (Schall et al., 2018). This improvement in overall mood and wellbeing in individuals with dementia has been supported through other studies involving artistic interventions (Belver et al., 2018). Additionally, engaging in art practices such as the visual arts helps cancer patients in meaning-making and building a positive identity (Stuckey and Nobel, 2010). Furthermore, art activities foster hopefulness, and facilitates recovery in people with mental illness (Stickley et al., 2018). Overall, it appears that art as an intervention (either passively or through active engagement) can benefit individuals in a clinical setting.

While the majority of the studies conducted on the arts assess its impact on human physiology and psychology within the clinical settings, there are several studies evaluating arts-based interventions to improve mental health and wellbeing in the non-clinical populations. These studies offer a promising potential for the future of research on how visual arts impact the general population. Within the general population, individuals who engaged with the arts for 100 or more hours annually had significantly higher mental wellbeing than others with less engagement (Davies et al., 2015). This artistic engagement may extend past the unimodal system of consumption, as shown by Stratton and Zalanowski (1989) who found that in combining the experience of paintings and music, which either prompted depression, positive affects, or neutrality, a participant’s mood could be influenced, whereas each medium by itself did not induce such effects. As is relevant in a pandemic context, an artistic virtual reality tool was shown to be effective in improving participants’ mood (Habak et al., 2020). Given the apparent beneficial effects of artistic interventions on mood, Scandinavian countries such as Sweden and Norway have developed a wide range of art programs which have been shown to improve mood, mental health, and overall wellbeing in their population (Jensen et al., 2017).

Given these findings, the purpose of this study was to investigate whether a virtual arts platform would improve the mood of attendees during the COVID-19 pandemic. The primary hypothesis was that negative affective scores would decrease, and positive affective scores would increase following the attendance of a virtual art exhibit. The secondary hypothesis was that the addition of music to the art exhibit would elicit a greater decrease in negative emotion and a greater increase in positive emotion.

## Materials and methods

### The project’s inception

The initial plan for this project in 2014 was an immersive experience where visitors could jointly observe visual arts in a gallery while listening to the corresponding pieces of music. However, due to the COVID-19 pandemic in 2020, this project was altered to align with the restrictions on people’s gatherings and public spaces, which proved to be a challenging task because of technological complexities and hurdles in creating a user-friendly yet realistic experience as well as visitors’ requirements to have stable internet connection and follow detailed instructions. Eight pieces of music were selected for the project and 28 artists were instructed to create visual works representing two out of the eight pieces of music. The artworks were shown in two formats, immersive and non-immersive. The two formats were identical except for the presentation of music in the immersive format.

### Artist recruitment and procedure

Artists (*n* = 28) were recruited for participation in the project from around the world through a referral basis and through searches on the Internet, online galleries, and museums. Each artist signed an informed consent form which included a description of the project, instructions, and additional information. Artists were selected from different countries (Canada = 9, Iran = 9, United Kingdom = 2, Netherlands = 1, Serbia = 1, Ukraine = 1, Denmark = 1, Germany = 1, Ghana = 1, South Africa = 1, Japan = 1) different education levels (Academic education in arts = 17, Private education = 4, High school art education = 1, self-taught = 6) and different musical background (No musical training = 11, beginner/intermediate level = 10, advanced/self-taught musician = 7). Only one artist had synesthesia. The participating artists were asked to visualize two different musical pieces into two works of visual art, to translate what they hear into what can be seen. The musical pieces were selected among a pool of 100 pieces (initially selected by SH) from various cultures and genres specifically for their emotive characteristics. Out of the selection of eight pieces chosen for this study, the artists were each randomly assigned one musical piece as per the researcher’s discretion and then asked to select a second piece from the rest of the list of eight pieces provided to them.

**FIGURE 1:**
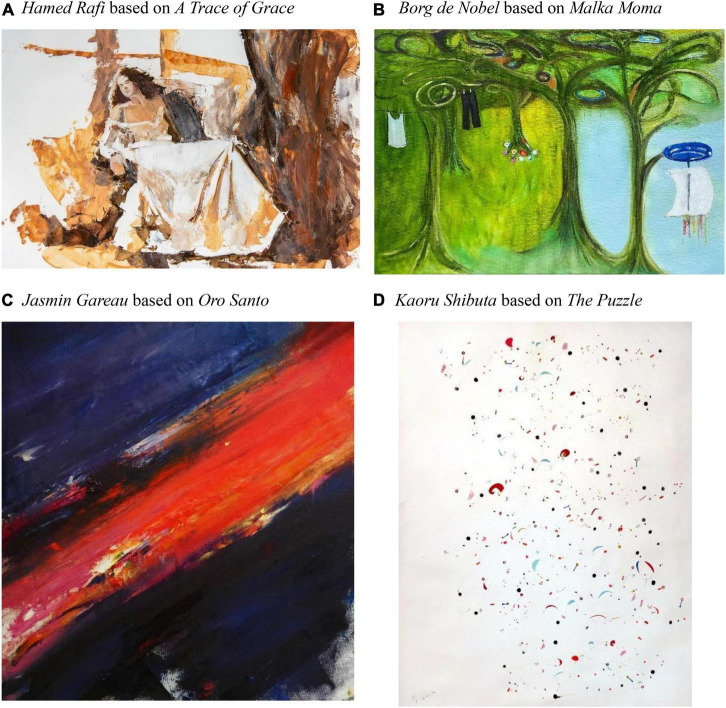
Excerpts of projects created for VisualEars exhibit and room. **(A)** Hamed Rafi based on A Trace of Grace. **(B)** Borg de Nobel based on Malka Moma. **(C)** Jasmin Gareau based on Oro Santo. **(D)** Kaoru Shibuta based on The Puzzle. Source: www.visualearsproject.com. Reproduced with permission.

The musical pieces selected from different musical cultures for this project were *Avminnast* by Nils Økland, *A Trace of Grace* by Michel Godard, Alim Qasimov, Rauf Islamov, and Hüsnü Şenlendirici (for example, see Figure 1A), *Malka Moma si se bogu moli* by Neli Andreeva and Georgi Genov (for example, see Figure 1B), Etude for Piano in C Sharp minor Op. 2, No 1. by Alexander Scriabin performed by Vladimir Horowitz, *Oro Santo* by Javier Limón featuring Buika (for example, see Figure 1C), *A Place for Us* by Brian Weafer, *The Puzzle* by Dawn Davi (for example, see Figure 1D) *Elegy for Viola* by Peter Cavallo. Out of these 8 pieces, five were instrumental, and three featured vocals. Artists were compensated with $ 50 (CAD) per work of art according to the CARFAC (Canadian Artists’ Representation) guidelines in Canada.

## Materials and measures

### Positive and negative affects survey (PANAS)

The positive and negative affects questionnaire (PANAS) (Watson et al., 1988) originally developed and validated as a 20-item measurement. PANAS is used to measure the changes in positive and negative effects of an individual and has been shown to be reliable (Positive Affect Scale, Cronbach’s α = 0.88; Negative Affect Scale, Cronbach’s α = 0.87; Watson et al., 1988). For this project, the English version as well as the French and Farsi (Shokri et al., 2014) translations were used. The version in Farsi shows comparable reliability within positive affects (Cronbach’s α = 0.83) and negative affect (Cronbach’s α = 0.78) dimensions (Shokri et al., 2014). Within our sample, the PANAS scale was sufficiently reliable within the English (Positive, Cronbach’s α = 0.92; Negative, Cronbach’s α = 0.94) and Farsi (Positive, Cronbach’s α = 0.84; Negative, Cronbach’s α = 0.85) translations. Alternatively, the French version should be interpreted with caution, due to the small sample size, individual evaluation of the scales was not possible, and 3 items (Item 7, 17, and 19) had to be removed due to lack of variance. Nonetheless, reliability was relatively adequate within the French translation of the PANAS within our small sample (Cronbach’s α = 0.93).

### Virtual exhibition

The exhibition was designed in two formats (immersive and non-immersive) on Kunstmatrix platform which hosts virtual 3D exhibitions that allow the visitors to interact closely with the visual arts hung on the walls, simultaneously listen to the music selected by the curator, and navigate the space by clicking on directional arrows. The two formats of the exhibition consist of a non-immersive version where there were artworks on the walls while the immersive version also included the musical piece which the visual arts were based upon. Therefore, in each room of the immersive format, the piece of music based on which the works of art were created was playing while the visitors were viewing the works of art. Both immersive and non-immersive versions consisted of eight separate rooms which were linked and navigated by clicking on designated buttons. Each room was slightly different in terms of the color of the walls and the layout in order to give the visitors a realistic impression of a physical exhibition. The exhibition and the study were live for 4 weeks. The participants were only told that they would be visiting an art exhibit that featured 8 rooms, where there would be just the visual works of art or visual arts pieces and music playing in the rooms. However, upon clicking on the description of each artwork, participants would be informed that each work of art was a visualization of a musical piece with the title of the piece next to it. In addition, by using newly created art, there was zero chance that the participants would be viewing works with which they were familiar. Thus, the visual aspect of the project was all “new” to all participants and therefore a kind of neutralizer.

It is noted that the designs of the immersive and non-immersive versions were identical therefore keeping music the only variable for the visitors. The rooms were to be visited consecutively and in one sitting through the instructions provided upon entering the first room and the same instructions offered in the first and last panels of each room. Rooms were chosen for each musical piece where the pieces of artwork were displayed that were made for that piece, therefore, there were eight rooms in total for the exhibition, the number of artworks in each room varies as some of the pieces were selected by more artists. There was one room that held only two artworks and there were a few rooms that held nine artworks.

### Experimental paradigm

The exhibition was launched on a dedicated website, www.visualearsproject.com and invitations were sent out to the general public through emails and instagram posts.

Participants were directed from the website to the study on Qualtrics through a link. The paradigm was designed on Qualtrics in three languages: English, French, and Farsi. Participants were asked to choose a language to complete the study and fill out an informed consent form. They were then directed to complete a pre-experience PANAS and follow by selecting their age range from three options of below 25, 25 to 50, and over 51. They were also asked to choose their gender from the categories male, female, and other.

Only participants who appropriately indicated their consent were directed to complete the study, if participants indicated dissent, they were invited to visit the immersive version without participation in the study. After the completion of the PANAS scale, participants were directed to the first room of the exhibition and were randomly assigned to either the immersive condition (music and visual) or to the non-immersive condition (visual only). Through the instructions, participants would enter room 1, and start from a panel that displayed the words “Start here.” They could click on the panel and then navigate the room by clicking to the artwork on their right side. In the immersive version, once the visitor clicked on the first panel, the music started playing and kept looping until they left the room. Each artwork came with the name of the artist, the piece of music which it was visualized upon, the price, the technique and size of the artwork, and the artist’s website. The visitors could zoom in on the artworks and manually navigate the room instead of using the right and left directive arrows on the page and leave for the next room by clicking on the link provided through the last panel in each room. Participants were asked to visit the exhibition and complete the relevant surveys in one sitting. In the last room, there was a panel through which participants were directed to take the post-experience PANAS once their visit was completed. The questionnaire was a duplicate of the first one followed by a question that asked visitors to describe their exhibition experience in one word.

### Statistical analyses

Overall composite affective emotion scores were calculated for positive and negative affective mood from the PANAS in English, French, and Farsi by calculating the average from 10 sub-categories. The composite positive affective emotion score was calculated from the following sub-categories: Interested, Excited, Strong, Enthusiastic, Proud, Alert, Inspired, Determined, Attentive, and Active. The composite negative affective emotion score was calculated from the following sub-categories: Distressed, Upset, Guilty, Scared, Hostile, Irritable, Ashamed, Nervous, Jittery, and Afraid. If participants were missing more than two sub-category responses from the respective composite score the individual was dropped from analyses. To investigate whether demographics (i.e., age, language, or gender), or experimental group assignment had any effect on completion of the art exhibit and post PANAS χ^2^ tests were conducted.

For the primary hypothesis linear mixed effects models (LME) with mood (i.e., composite positive affective emotion score), with (i) fixed effects of age, gender, music, and time (i.e., before vs. after the virtual exhibit); (ii) random effects of intercept and slope of time by subject and (iii) a first order autoregressive covariance matrix (i.e., corAR1) between time points. For the secondary hypothesis investigating moderators of the effect that attending a virtual art exhibit has on mood an additional fixed effect of the interaction between time with either age, gender, or music was included [equation: Mood ∼ Age + Gender + Time + Group + (∼ 1 + Time | Subj), correlation = corAR1]. Additional exploratory LME analyses for each sub-category within each composite affective score were conducted. To correct for multiple comparisons a false discovery rate (FDR) using the two-stage linear step-up procedure by Benjamini et al. (2006) was applied in a family wise basis within the (i) primary hypothesis (i.e., correcting for testing the composite positive and negative score); (ii) secondary hypothesis; and (iii) exploratory hypothesis (i.e., correcting for 10 sub-categories with each composite score). All statistical analyses were conducted using R 4.0.5. Unstandardized beta values were reported as measures of effect sizes for all findings.

## Results

### Participants

**FIGURE 2:**
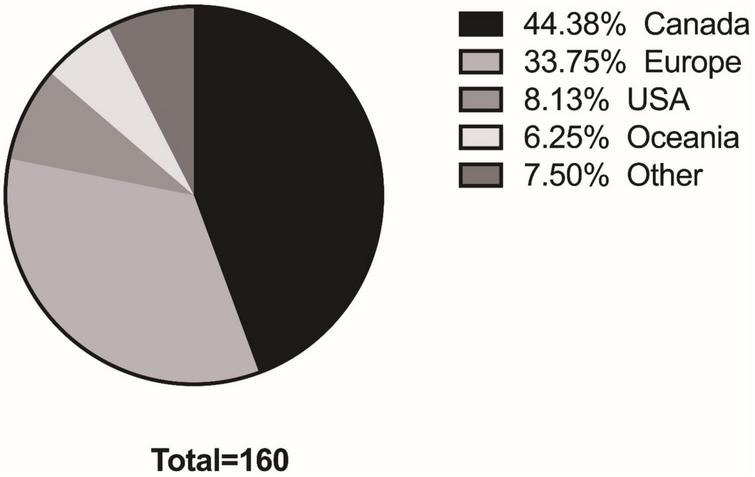
Participant’s locations.

Participants (*n* = 160) initially completed a pre-experience survey consisting of basic demographic information surrounding participants’ gender, age category, and language choice for completing surveys. Participants locations were widely spread across the world as shown in Figure 2. Participants were randomly assigned into an experimental group (*n* = 80), or a control group, with significantly more individuals selecting the English version, than the Farsi or French; this distribution saw significant change between experimental and control groups from pre- vs. post- experience [χ^2^(4) = 61.17, *p* < 0.001]. Following the immersive art experience, participants (*n* = 60, experimental = 33) answered the post-experience survey. Participants attrition between pre- and post- survey varied by age, gender, and language (see Table 1). While considerable attrition was observed from pre- to post- experience survey, no significance differences were found in relation to recorded demographic variables between those who completed the pre-survey and those who completed the post-survey, in regard to the sample’s gender [χ^2^(4) = 2.42, *p* = 0.66], age categories [χ^2^(4) = 4.52, *p* = 0.34], choice of language [χ^2^(4) = 0.66, *p* = 0.96], or group type [χ^2^(1) = 0.60, *p* = 0.43].

**TABLE 1:** Demographic information for participants pre–and post–immersive experience.

Time-Point	Group	Gender	Language	Age
Pre-experience	Experimental (*n* = 80)	Female = 50	EN = 68	<25 = 6
		Male = 28	FR = 3	25–50 = 48
		Other = 1	FA = 9	>50 = 26
		Undisclosed = 1		
	Control (*n* = 80)	Female = 49	EN = 64	<25 = 4
		Male = 28	FR = 1	25–50 = 45
		Other = 2	FA = 15	>50 = 31
		Undisclosed = 1		
Post-experience	Experimental (*n* = 33)	Female = 18	EN = 27	<25 = 2
		Male = 15	FR = 1	25–30 = 14
			FA = 5	>50 = 17
	Control (*n* = 27)	Female = 14	EN = 21	<25 = 2
		Male = 12	FR = 0	25–50 = 13
		Other = 1	FA = 6	>50 = 12

EN, English; FA, Farsi; FR, French.

### Demographic characteristics and mood

**FIGURE 3:**
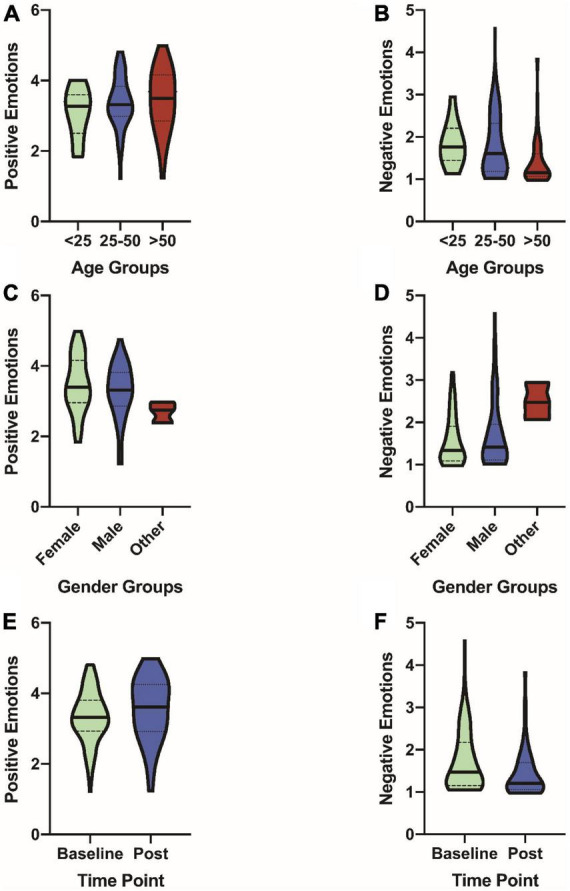
Violin plots of demographics and time with PANAS scores. **(A)** Age vs. positive emotions. **(B)** Age vs. negative emotions. **(C)** Gender vs. positive emotions. **(D)** Gender vs. negative emotions. **(E)** Time vs. positive emotions. **(F)** Time vs. negative emotions.

The association of age and sex with PANAS scores are presented in Table 2. Older participants had significantly lower composite scores of negative affective emotions (*b* = −0.38, p_*FDR*_ = 0.002; Figure 3B), and its sub-category questions: Distressed (*b* = −0.47, p_*FDR*_ = 0.003), Upset (*b* = −0.33, p_*FDR*_ = 0.04), Guilty (*b* = −0.52, p_*FDR*_ = 0.0004), Ashamed (*b* = −0.31, p_*FDR*_ = 0.02), Nervous (*b* = −0.45, p_*FDR*_ = 0.005), Afraid (*b* = −0.31, p_*FDR*_ = 0.03). Additionally, while age was not significantly associated with the composite positive affective emotion (*b* = 0.10, p_*FDR*_ = 0.20; Figure 3A), older participants had higher positive affective emotion in the sub-categories: Alert (*b* = 0.40, p_*FDR*_ = 0.03), Attentive (*b* = 0.33, p_*FDR*_ = 0.03). Male participants had a significantly lower composite score of positive affective emotion (*b* = −0.22, p_*FDR*_ = 0.04; Figure 3C), and higher composite score of negative affective emotion (*b* = 0.26, p_*FDR*_ = 0.02; Figure 3D) and its sub-category questions: Scared (*b* = 0.35, p_*FDR*_ = 0.04), and Hostile (*b* = 0.44, p_*FDR*_ = 0.002).

**TABLE 2:** Results of linear mixed effects analyses investigating the association of time, age and gender with positive and negative affect.

Measure	Mean ± SD		Predictors								
	Mean score pre	Mean score post	Time			Age			Sex		
	*n* = 159	*n* = 58	b	*P*	pFDR	P	*p*	pFDR	b	*P*	pFDR
Positive	3.32 ± 0.84	3.54 ± 0.98	0.17	0.03	0.03*	0.10	0.38	0.20	–0.22	0.08	0.04*
Negative	1.72 ± 0.79	1.43 ± 0.66	–0.19	0.01	0.02*	–0.31	< 0.001	0.002*	0.26	0.02	0.02*
Interested	3.92 ± 1.04	3.83 ± 1.19	–0.08	0.50	0.45	0.06	0.66	0.53	–0.26	0.10	0.20
Distressed	1.94 ± 1.1	1.55 ± 0.9	–0.28	0.02	0.06	–0.47	< 0.001	0.003*	0.31	0.04	0.11
Excited	3.15 ± 1.15	3.26 ± 1.22	0.12	0.34	0.34	–0.12	0.43	0.39	–0.16	0.35	0.39
Upset	1.77 ± 1.14	1.52 ± 0.98	–0.18	0.16	0.25	–0.33	0.02	0.04*	0.14	0.38	0.40
Strong	3.04 ± 1.14	3.17 ± 1.29	0.06	0.58	0.49	0.14	0.37	0.36	–0.30	0.08	0.19
Guilty	1.59 ± 0.96	1.33 ± 0.89	–0.10	0.34	0.34	–0.52	< 0.001	0.0004*	0.30	0.02	0.10
Scared	1.59 ± 0.92	1.29 ± 0.65	–0.24	< 0.001	0.03*	–0.19	0.07	0.10	0.35	< 0.001	0.04*
Hostile	1.43 ± 0.82	1.26 ± 0.66	–0.11	0.19	0.26	–0.13	0.21	0.24	0.44	< 0.001	0.002*
Enthusiastic	3.37 ± 1.11	3.41 ± 1.2	0.07	0.50	0.45	< 0.001	0.99	0.67	–0.18	0.29	0.34
Proud	3.11 ± 1.31	3.47 ± 1.52	0.43	0.01	0.03*	–0.07	0.71	0.54	–0.28	0.15	0.22
Irritable	1.92 ± 1.15	1.57 ± 0.86	–0.25	0.02	0.06	–0.16	0.28	0.29	0.07	0.67	0.64
Alert	3.18 ± 1.21	3.53 ± 1.25	0.13	0.33	0.34	0.40	0.01	0.03*	–0.07	0.68	0.64
Ashamed	1.47 ± 0.88	1.24 ± 0.6	–0.10	0.07	0.15	–0.31	< 0.001	0.02*	0.30	0.01	0.08
Inspired	3.34 ± 1.22	3.84 ± 1.31	0.47	< 0.001	0.03*	0.08	0.64	0.53	–0.35	0.05	0.14
Nervous	1.93 ± 1.03	1.59 ± 1.03	–0.25	0.03	0.08	–0.45	< 0.001	0.005*	0.22	0.14	0.21
Determined	3.29 ± 1.17	3.6 ± 1.32	0.21	0.14	0.23	0.21	0.18	0.23	–0.28	0.10	0.20
Attentive	3.55 ± 0.99	3.86 ± 1.13	0.15	0.22	0.28	0.33	0.01	0.03*	–0.16	0.27	0.34
Jittery	1.91 ± 1.06	1.53 ± 0.96	–0.27	0.01	0.04*	–0.27	0.06	0.09	0.18	0.24	0.33
Active	3.24 ± 1.05	3.41 ± 1.06	0.18	0.08	0.15	0.01	0.94	0.67	–0.25	0.11	0.20
Afraid	1.60 ± 0.99	1.41 ± 0.92	–0.14	0.27	0.33	–0.32	0.01	0.03*	0.31	0.03	0.10

*P-values significant at α = 0.05.

### Effect of the virtual art exhibit on mood

The effect of attending a virtual art exhibit (i.e., Time) on mood questionnaire scores are presented in Table 2. Following the virtual art exhibit attendees had a higher composite score of positive affective emotion (*b* = 0.17, p_*FDR*_ = 0.03; Figure 3E), and its sub-category questions: Proud (*b* = 0.43, p_*FDR*_ = 0.03), and Inspired (*b* = 0.47, p_*FDR*_ = 0.03). Additionally, following the virtual art exhibit attendees had a lower composite score of negative affective emotion (*b* = −0.19, p_*FDR*_ = 0.02; Figure 3F), and its sub-category questions: Scared (*b* = −0.24, p_*FDR*_ = 0.03), and Jittery (*b* = −0.27, p_*FDR*_ = 0.04).

### Moderators of the virtual art exhibit on mood

The moderating effects of incorporating music into some participant’s virtual art exhibit, age and gender are presented in Table 3. Age was a significant moderator of the effect that attending a virtual art exhibit has on mood. Younger participants had a greater increase in the composite positive affective score (*b* = −0.29, p_*FDR*_ = 0.02). and its sub-category: Inspired (*b* = −0.67, p_*FDR*_ = 0.04) than older participants. Younger participants had a greater decrease in the composite negative affective score (*b* = 0.29, p_*FDR*_ = 0.02). and its sub-category: Hostile (*b* = 0.42, p_*FDR*_ = 0.04) and Irritable (*b* = 0.49, p_*FDR*_ = 0.04) than older participants. Neither the incorporation of music alongside the virtual art exhibit or gender were significant moderators of the effect of a virtual art exhibit on mood.

**TABLE 3:** Results of linear mixed effects analyses investigating the moderating effects of group, age and gender on the change in positive and negative affect from attending the virtual art exhibit.

Measure	Moderators of response								
	Group			Age			Sex		
	b	*P*	pFDR	b	*P*	pFDR	P	*p*	pFDR
Positive	–0.11	0.46	0.82	–0.29	0.02	0.02*	–0.06	0.71	0.75
Negative	–0.04	0.78	0.82	0.29	0.01	0.02*	0.23	0.09	0.20
Interested	–0.44	0.07	0.93	–0.28	0.17	0.27	0.01	0.98	>0.99
Distressed	–0.22	0.34	0.93	0.31	0.12	0.24	0.20	0.40	0.69
Excited	–0.41	0.10	0.93	0.16	0.46	0.46	0.45	0.08	0.60
Upset	–0.19	0.46	0.93	0.28	0.19	0.28	0.30	0.24	0.69
Strong	0.23	0.31	0.93	–0.14	0.47	0.46	–0.10	0.65	0.98
Guilty	0.13	0.51	0.93	0.31	0.07	0.24	0.22	0.27	0.69
Scared	0.01	0.93	>0.99	0.20	0.14	0.26	0.01	0.93	>0.99
Hostile	0.09	0.58	0.93	0.42	< 0.001	0.04*	0.02	0.89	>0.99
Enthusiastic	–0.14	0.54	0.93	–0.15	0.43	0.46	< 0.001	0.99	>0.99
Proud	0.09	0.77	>0.99	–0.09	0.73	0.69	–0.08	0.78	>0.99
Irritable	0.17	0.43	0.93	0.49	0.01	0.04*	0.24	0.27	0.69
Alert	–0.26	0.33	0.93	–0.34	0.12	0.24	–0.23	0.38	0.69
Ashamed	0.04	0.70	0.98	< 0.001	0.98	0.88	0.12	0.29	0.69
Inspired	–0.07	0.82	>0.99	–0.67	< 0.001	0.04*	–0.32	0.27	0.69
Nervous	–0.15	0.51	0.93	0.15	0.44	0.46	0.40	0.08	0.60
Determined	< 0.001	1.00	>0.99	–0.36	0.12	0.24	–0.27	0.33	0.69
Attentive	< 0.001	1.00	>0.99	–0.35	0.08	0.24	–0.19	0.43	0.69
Jittery	–0.09	0.66	0.98	0.17	0.31	0.43	0.57	< 0.001	0.08
Active	–0.28	0.16	0.93	–0.14	0.40	0.46	–0.02	0.91	> 0.99
Afraid	–0.26	0.30	0.93	0.41	0.05	0.22	0.38	0.13	0.67

SD, standard deviation; b, unstandardized beta estimate; *P-values significant at α = 0.05.

## Discussion

The goal of this novel research project was to find out the impact of a virtual art exhibition incorporating music and visual arts on the positive and negative affects of the general public during the COVID-19 pandemic. As in-person human interaction was limited, the pandemic took its toll on the mental wellbeing of millions around the globe, in particular, the general public and those without any known history of mental health challenges. A Dutch longitudinal study revealed that people without a mental illness showed a greater increase in symptoms during the COVID-19 pandemic as opposed to those with a mental illness (Pan et al., 2021). Although this project was initially conceived to take place in person, COVID-19 created an opportunity for a widespread audience.

The exhibition in this study took place in two formats, one of which contained only visual works of art, and the other contained music and visual artworks. PANAS was utilized to evaluate the positive and negative affects of the audience anonymously before and after the virtual exhibition through a randomized and controlled experiment. The study ran for 4 weeks and was accessed by a widespread audience in three languages, English, French, and Farsi. The subjects were asked to rank their positive and negative affects on a scale from 1 to 5. Each format of the exhibition consisted of 8 rooms each of which hosted a number of artworks which were visualizations of a particular musical piece. The immersive format also featured the musical pieces in each room. The rooms were visited consecutively by the audience who were randomly assigned the immersive or the non-immersive format.

This study demonstrated that a virtual art exhibition during the pandemic was able to increase the positive affect and decrease negative affect in a widespread audience. Furthermore, younger participants benefited more (i.e., higher increase in positive affect and greater decrease in negative affect) than older participants. Given the technological aspect of participating in a virtual platform, it is possible that the greater benefit observed in younger participants may be attributed to higher technological fluency in this group. Research has demonstrated younger participants frequently and fluently use computers and other technology for various tasks in order to work, socialize, study, and etc. (Olson et al., 2011). Nonetheless, prior studies by Davies et al. (2015) and Habak et al. (2020) have found that a virtual reality tool and engagement in art was associated with better mood. Furthermore, the current study supports findings from the project “Art and Wellbeing” involving four European institutions that found that “Receptive participation in the arts (visual arts, theater, dance, architecture and heritage) during the pandemic was significantly correlated to a decrease in negative feelings.” Additionally, a study by Ascolani et al. (2020) indicates that 85.18% of the participants consumed different forms of art as a coping mechanism, and 64.21% declared that art makes them feel better. Therefore, suggesting that engagement in the arts may serve as a beneficial and widely accessible intervention to improve mood during a pandemic.

Interestingly our study found that older participants had lower overall negative affect, including its subcategories of distress, upset, and nervousness. Older participants also reported being more alert and attentive which are subcategories of overall positive affect. Furthermore, Male-identifying subjects had a significantly lower overall positive affect and higher overall negative affect, including its subcategories of scared and hostility. A recent study by Fenollar-Cortés et al. (2021) that also used the PANAS found that during the midst of lockdowns older participants had significantly higher positive affect and nominally lower negative affect. These results also align with another study which found that the negative psychological impact of COVID-19 pandemic hits young people harder (Justo-Alonso et al., 2020). These results might also reveal that older participants were coping better with the negative impacts of the pandemic. Regarding gender differences in affect, future research is needed as some studies find no differences (Fenollar-Cortés et al., 2021) while other studies have reported that females are hit harder by the negative psychological impact of COVID-19 pandemic (Justo-Alonso et al., 2020).

Although the initial hypothesis of the VisualEars project was that subjects visiting the rooms with music and visual artworks would show a greater increase in the positive affects and decrease in the negative affects relative to those visiting the rooms with the visual artworks only, music did not appear to impact the results. This contrasts with a study by Stratton and Zalanowski (1989) that showed that the combination of viewing paintings and listening to music altered the mood of the participants, whereas music and paintings separately did not. Although the methods of this study differ from the VisualEars project, future research on the simultaneous pairing of art and music is warranted. Some of the challenges in this study and future opportunities are outlined below:

Firstly, technical challenges could most likely account for the hindrances, limitations, and the lack of support for the hypothesis. This virtual art exhibit was made possible through connecting three different platforms: a dedicated website to the project as a means to introduce and begin the experiment, Qualtrics on which the consent forms and the questionnaires were held, and Kunstmatrix where the 3D exhibition was offered. Although every effort was made to make the experience as seamless as possible, it would be remiss to consider it an easy-to-navigate for all. For example, the instructions were given on navigating the exhibition for best audio results. Each room would open in a new tab, and the audience were advised to close the previous tab right after opening a new tab while moving from room to room. However, some visitors reported not reading the instructions and therefore, encountered instances when multiple musical pieces were playing simultaneously. They were also directed to use their computers, the Chrome browser, and headphones while visiting to allow for the best experience; however, there were anecdotes of participants visiting on their phones or listening to the music on speakers due to lack of access to a computer or a working headphone. Additionally, in order to create a smooth flow, the rooms were designed in a way that would not allow visitors to go back to a room, hence if a visitor accidentally closed their tab, they would have needed to start over and lose their initial consent form and questionnaire responses. Another obstacle was the bandwidth issues on some visitors’ end and some internal server interruptions which happened to Qualtrics and Kunstmatrix during the time the exhibition was live. These problems resulted in many visitors not being able to follow the rooms and finish their visit as designed and expected. Therefore, the result of the positive and negative affects scores are likely to be more favorable than the current outcome and it is desirable to conduct the study again applying other platforms or at a designated lab to prevent external obstacles. It would also be crucial to run this study on a larger pool of participants to examine the further implications as a means to temporarily enhance and regulate mood as an accessible tool for self-care.

Secondly, unfamiliarity with the genres of the musical selections might have prevented some participants from connecting with the music. In addition, some participants with preference for more upbeat music might have not connected with the pieces employed in this study as all pieces were more reflective and contemplative in mood and tended to have slower tempos.

Thirdly, the three pieces of music which featured vocals in Bulgarian, Spanish, and Azerbaijani languages may have caused an unintentional disconnect between the musical piece and the visual work of art and therefore preventing the immersive format to elicit better results in terms of mood regulation for the participants. There are two potential reasons here. (1) The artists if not fluent in the language of the music piece may have envisioned it differently. (2) If participants didn’t understand the language the piece was composed in, that may have impaired them connecting the art to music in the way the artist intended.

A few suggestions are noted concerning future replications of this study. (1) There were instructions on the first panel of the exhibit on how to navigate each room from start to the end for a more seamless experience, but participants were free to navigate it the way they wanted as well using the zooming in and out option, and the arrows to navigate in different directions; however, the order of the works and rooms were fixed. This order was one which the designing author thought would be aesthetically pleasing. We acknowledge that it might have had a specific impact on the audience and we suggest that the order of the artworks randomly change for each participant in any future replications of this study. (2) The fact that the visitors chose to attend the art exhibit revealed their willingness and openness to engage in an artistic activity which happened to be a novel experience as well. These participants might have been more likely to have positive experiences from an art exhibit and therefore might not have represented the general public. We suggest further data collection about each participant’s level of engagement with artistic activities and the application of further scales such as Sandstrom and Russo’s (2013) AIMS (Absorption in Music Scale) prior to the study to illuminate the correlation if any with the outcome of the research. (3) We strongly recommend a replication of this study with a simpler architectural design for the exhibit and reliance on simple elements of technological fluency.

In regard to the future opportunities, developing and expanding a dedicated smartphone and web application for this project can serve as a self-care tool for the majority of the general population, in particular adolescents and young people since significant improvement was observed in their age group. Furthermore, the results of this study warrant a study in clinical settings in Toronto such as CAMH, The Baycrest, The Alzheimer’s Society and other mental health organizations in order to evaluate this mood enhancing intervention for their clientele. Amidst the growing increase in mental health problems especially in more vulnerable and marginalized populations, such exhibitions can offer a glimmer of hope to help with mood enhancement and regulation as alternative and temporary interventions. Since the PANAS results showed significant changes in the positive and negative affects of the general population, VisualEars may also be an accessible intervention for addressing the sub-clinical increase in anxiety and stress due to COVID-19 and other similar crises.

Streaming links to musical pieces used in this study are found below. It is noted that the digital recordings of these pieces were used in the exhibit with the permission of the artists and producers.^1, 2, 3, 4, 5, 6, 7, 8^

## Data availability statement

The data that support the findings of this study are available on request from the corresponding author. The data are not publicly available due to privacy or ethical restrictions.

## Ethics statement

The studies involving human participants were reviewed and approved by the REB York University. The patients/participants provided their written informed consent to participate in this study. This study was approved by the Certificate #: STU 2021-044 (Approval Period: April 30, 2021–2022).

## Author contributions

SH designed, conceived of the experiment, and created the online interface. KK and GM conducted statistical analysis. JD supervised the entire process. All authors contributed to the article and approved the submitted version.

## Footnotes

1
https://open.spotify.com/track/2HuURRB0fsuPmU27NKikRg?si=8c094289e3c74924

2
https://open.spotify.com/track/2uiyg1Wo0f5NwHhblrf5yb?si=a358030836444c2f

3
https://open.spotify.com/track/7utrZiOvt4WzxK0wUjE1Ze?si=7bdc70ea97cb4795

4
https://open.spotify.com/track/0yU2bE32QLoEt6BWqhRJVz?si=aaab2daca5514a09

5
https://open.spotify.com/track/1GbAJjK2MTp3TA5MNB8Efo?si=014a3c80f67c4716

6
https://open.spotify.com/track/068Ygx4bG8siAjLQo8t5rc?si=277f9ddf26554c21

7
https://music.apple.com/ca/album/malka-moma-si-se-bogu-moli/907819024?i=907819082

8
https://www.youtube.com/watch?v=cHkXM-i_1MU
